# Corrigendum to a cross-industry collaboration to assess if acute toxicity (Q)SAR models are fit-for-purpose for GHS classification and labelling. Regulatory toxicology and pharmacology (2021) 104843

**DOI:** 10.1016/j.yrtph.2022.105165

**Published:** 2022-04-23

**Authors:** Joel Bercu, Melisa J. Masuda Herrera, Alejandra Trejo-Martin, Catrin Hasselgren, Jean Lord, Jessica Graham, Matthew Schmitz, Lawrence Milchak, Colin Owens, Surya Hari Lal, Richard Marchese Robinson, Sarah Whalley, Phillip Bellion, Anna Vuorinen, Kamila Gromek, William A. Hawkins, Iris Van de Gevel, Kathleen Vriens, Raymond Kemper, Russell Naven, Pierre Ferrer, Glenn J. Myatt

**Affiliations:** a Gilead Sciences, 333 Lakeside Drive, Foster City, CA, USA; b Genentech, Inc., 1 DNA Way, South San Francisco, CA, 94080, USA; c Bristol Myers Squibb, Drug Safety Evaluation, 1 Squibb Dr, New Brunswick, NJ, 08903, USA; d AbbVie Inc., North Chicago, IL, USA; e 3M Company, 3M Center, St. Paul, MN, 55144-1000, USA; f Syngenta Crop Protection, Product Safety Department, Jealott’s Hill International Research Centre, Bracknell, Berkshire, RG42 6EY, UK; g DSM Nutritional Products, Kaiseraugst, Switzerland; h Galapagos SASU, 102 Avenue Gaston Roussel, 93230, Romainville, France; i GlaxoSmithKline, Park Road, Ware, Hertfordshire, SG12 ODP, United Kingdom; j Janssen Pharmaceutical Companies of Johnson & Johnson, 2340, Beerse, Belgium; k Vertex Pharmaceuticals Inc., Discovery and Investigative Toxicology, 50 Northern Ave, Boston, MA, USA; l Department of Veterinary Physiology and Pharmacology, Interdisciplinary Faculty of Toxicology Program, Texas A&M University, 4466 TAMU, College Station, TX, 77843-4466, USA; m Leadscope (an Instem Company), 1393 Dublin Rd, Columbus, OH, 43215, USA; n Ultragenyx, 60 Leveroni Court, Novato, CA, 94949, USA

The study presented in Bercu et al. (2021) was designed to understand whether (Quantitative) Structure-Activity Relationship ((Q)SAR) models are fit-for-purpose to use as part of classification and labelling. To test this hypothesis, proprietary and marketed data on rat oral acute toxicity from 10 organizations representing chemicals typically assessed was compiled and run through (Q)SAR models developed by Leadscope (an Instem company). The experimental and prediction data from all collaborators was then combined and an assessment of whether these models are fit-for-purpose was made based on their performance. In addition, an expert review was performed and documented on a subset of the chemicals. Based on this information, a decision tree was presented that showed how these models could be used to support classification and labelling decisions. A reassessment of the results from one of the collaborators was recently performed because: (a) some compounds were identified as belonging to the (Q)SAR training sets; (b) some compounds were found to be duplicates following computation of InChIs; (c) integration with a more highly curated set of experimental data led to the experimental labels being updated for some compounds. The revised results were then combined with other collaborators’ results. The revised results do not change the conclusions or recommendations of the paper based on both the overall and subset specific balanced statistics for the consensus predictions. For example, the original abstract stated that approximately 95% of chemicals were either correctly predicted or predicted in a more conservative GHS category, after removing a small fraction of inconclusive - meaning indeterminate or out of domain - predictions. In the original analysis this value was 94.82% whereas in the revised analysis the figure is 94.84%. Similarly, in the original analysis, the average percentage of these compounds, across all well-defined experimental categories, which were assigned to a correct or more conservative category was around 80%. Excluding the two GHS category 1 compounds, since two compounds are too few to obtain robust statistics, the average percentage of these compounds which are assigned to a correct or more conservative category is 78%. The following figures and tables have been updated to reflect these changes: Fig. 4, Tables 2–6, supplemental materials Tables s1–s22. These figures and tables are included in the supplemental material.

## Supplementary Material

1

## References

[R1] Bercu J, Masuda-Herrera MJ, Trejo-Martin A, Hasselgren C, Lord J, Graham J, Schmitz M, Milchak L, Owens C, Hari Lal S, Marchese Robinson R, Whalley S, Bellion P, Vuorinen A, Gromek K, Hawkins WA, van de Gevel I, Vriens K, Kemper R, Naven R, Ferrer P, Myatt GJ, 2021. A cross-industry collaboration to assess if acute oral toxicity (Q)SAR models are fit-for-purpose for GHS classification and labelling. Regul. Toxicol. Pharmacol 120, 104843 10.1016/j.yrtph.2020.104843. 33340644 PMC8005249

