# Supplementary material for: Corrigendum to a cross-industry collaboration to assess if acute toxicity (Q)SAR models are fit-for-purpose for GHS classification and labelling. Regulatory toxicology and pharmacology (2021) 104843
Source: Regul Toxicol Pharmacol. Author manuscript; Available in PMC 2022 Jun 15. (PMC9200224; doi:10.1016/j.yrtph.2022.105165)
Supplement: 1 [file NIHMS1804085-supplement-1.pdf]

Supplemental Material
Manuscript revisions

Table 2: Table showing counts of how the consensus model predicts for the different GHS categories

		Predicted[c]						
**Experimental**		**Cat. 1**	**Cat. 2**	**Cat. 3**	**Cat. 4**	**Cat. 5**	**NC**	**Total**
	**Cat. 1**	0[a]	1	0	0	1	0	2
	**Cat. 2**	1	8[a]	5	2	2	1	19
	**Cat. 3**	1	13	30[a]	31	2	1	78
	**Cat. 4**	2	24	88	163[a]	30	3	310
	**Cat. 5**	1	11	37	73	43[a]	9	174
	**Cat. 5 or NC[b]**	3	46	157	319	119[a]	19[a]	663
	**NC**	8	18	74	185	97	79[a]	461
**Total**		16	121	391	773	294	112	1,707

a: Indicates where a correct prediction is made

b: Where chemicals were identified as > 2,000 mg/kg they were placed in category "Cat. 5 or NC" and not in Cat.5 or NC

c: Not including inconclusive predictions

Table 3: Breakdown of the consensus model results across different categories

			Fit-for-purpose[b]		Accuracy[c]	
**Experimental value**	**Count**	**Number of inconclusive predictions[a]**	**Percentage correct or more conservative**	**Percentage correct or one more conservative**	**Percentage correct**	**Percentage correct (+/- one category)[d]**
**Cat. 1[e]**	2	0	0%	0%	0%	50.0%
**Cat. 2**	20	1	47.4%	47.4%	42.1%	73.7%
**Cat. 3**	80	2	56.4%	55.1%	38.5%	94.9%
**Cat. 4**	322	12	89.4%	81.0%	52.6%	90.7%
**Cat. 5**	182	8	94.8%	66.7%	24.7%	71.8%
**Cat. 5 or NC**	703	40	100.0%	68.9%	20.8%	68.9%
**NC**	496	35	100.0%	38.2%	17.1%	38.2%

a. Not included in the statistics

b. An assessment of whether the (Q)SAR test is fit-for-purpose for classification and labelling, that is it predicts either the correct or a more potent/conservative category (or predicts one category more potent/conservative)

c. An assessment of the accuracy of the (Q)SAR test, that is the proportion of correctly predicted or +/- one GHS category

d. For risk assessment it is more important to have a correct or more conservative prediction than one that predicts a lower category; however, this statistic was included for completeness.

e. Statistics included for consistency with analysis in original publication

Table 4: Balanced summary statistics result for the consensus model

	Fit-for-purpose		Accuracy	
	**Average[a] percentage correct or more conservative**	**Average[a] percentage correct or one more conservative**	**Average[a] percentage correct**	**Average[a] percentage correct (+/- one category)**
Consensus model	77.6%	57.7%	35.0%	73.8%
Random model	63.2%	30.5%	14.5%	46.3%

a. Averages across all experimental classes, excluding compounds in the "Cat. 5 or NC" class and cat. 1 since there were only two chemicals, which is not sufficient for a robust estimation.

Table 5: Table showing the results of an expert review of the consensus prediction, with the original consensus prediction results shown in parentheses

	**Predicted**						
**Experimental**	**Cat. 1**	**Cat. 2**	**Cat. 3**	**Cat. 4**	**Cat. 5**	**NC**	**Total**
**Cat. 1**	2 (0)	0 (1)	0 (0)	0 (0	0 (1)	0 (0)	2 (2)
**Cat. 2**	1 (1)	15 (8)	2 (5)	0 (2)	0 (2)	0 (1)	18[a] (19)

a - there is one compound less in the total column for GHS Cat.2 (i.e., 18 (19)) since one result (ID 703) was assigned to inconclusive after an expert review

Table 6: Performance metrics showing the results of an expert review of the consensus prediction, with the original consensus performance metrics without expert review shown in parentheses

			Fit-for-purpose		Accuracy	
**Experimental value**	**Count**	**Number of inconclusive results**	**Percentage correct or more conservative**	**Percentage correct or one more conservative**	**Percentage correct**	**Percentage correct (+/- one category)**
**Cat. 1**	2	0	100.0% (0%)	100.0% (0%)	100.0% (0%)	100.0% (50%)
**Cat. 2**	20	2	88.9% (47.4%)	88.9% (47.4%)	83.3% (42.1%)	100.0% (73.7%)

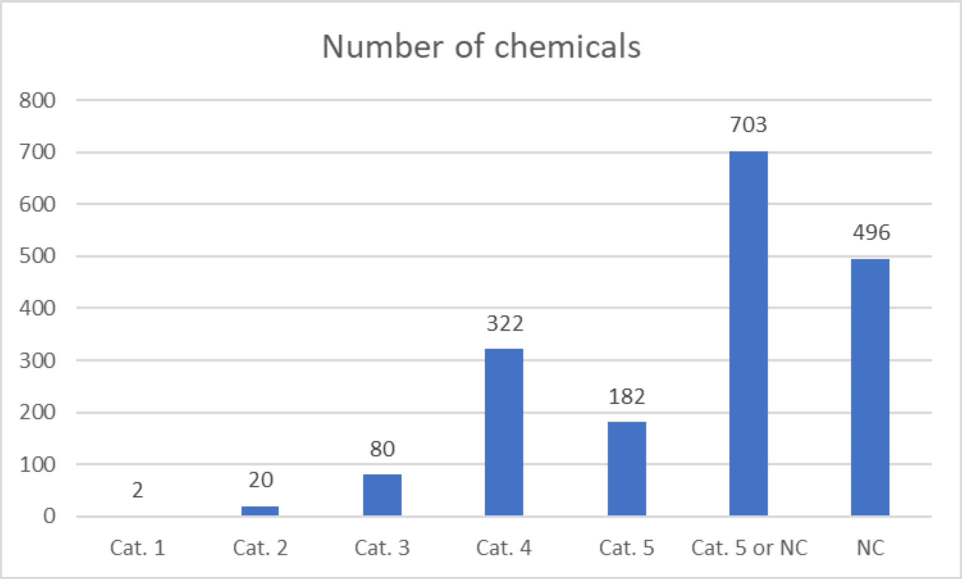

Figure 4: Number of chemicals for each experimental *in vivo* GHS category

Supplemental material revisions

1. Statistical-based Model

Table S1: Table showing counts of how the statistical-based model predicts for the different GHS categories

		Predicted[c]						
**Experimental**		**Cat. 1**	**Cat. 2**	**Cat. 3**	**Cat. 4**	**Cat. 5**	**NC**	**Total**
	**Cat. 1**	0[a]	1	0	0	0	1	2
	**Cat. 2**	1	5[a]	1	7	1	2	17
	**Cat. 3**	1	6	21[a]	35	6	0	69
	**Cat. 4**	2	20	28	175[a]	53	14	292
	**Cat. 5**	1	8	8	66	48[a]	21	152
	**Cat. 5 or NC[b]**	3	32	30	222	167[a]	111[a]	565
	**NC**	8	10	18	112	85	147[a]	380
**Total**		16	82	106	617	360	296	1,477

a: Indicates where a correct prediction is made

b: Where chemicals were identified as > 2,000 mg/kg they were placed in category "Cat. 5 or NC" and not in Cat.5 or NC

c: Not including inconclusive predictions

Table S2: Breakdown of the results across different categories for the statistical-based model

			Fit-for-purpose		Accuracy	
**Experimental value**	**Count**	**Number of inconclusive predictions[a]**	**Percentage correct or more conservative**	**Percentage correct or one more conservative**	**Percentage correct**	**Percentage correct (+/- one category)**
**Cat. 1**	2	0	0%	0%	0%	50.0%
**Cat. 2**	20	3	35.3%	35.3%	29.4%	41.2%
**Cat. 3**	80	11	40.6%	39.1%	30.4%	89.7%
**Cat. 4**	322	30	77.1%	69.5%	59.9%	87.7%
**Cat. 5**	182	30	86.2%	75.0%	31.6%	88.8%
**Cat. 5 or NC**	703	138	100.0%	88.5%	49.2%	73.2%
**NC**	496	116	100.0%	61.1%	38.7%	61.1%

a. Not included in the statistics

Table S3: Balanced summary statistics result for the statistical-based model

	Fit-for-purpose		Accuracy	
	**Average[a] percentage correct or more conservative**	**Average[a] percentage correct or one more conservative**	**Average[a] percentage correct**	**Average[a] percentage correct (+/- one category)**
Statistical-based model	67.8%	56.0%	38.0%	73.7%

a. Averages across all experimental classes, excluding compounds in the “Cat. 5 or NC” class and cat. 1 since there were only two chemicals which is not sufficient for a robust estimation.

b. Expert rule-based Model

Table S4: Table showing counts of how the expert rule-based model predicts for the different GHS categories

		**Predicted[c]**						
**Experimental**		**Cat. 1**	**Cat. 2**	**Cat. 3**	**Cat. 4**	**Cat. 5**	**NC**	**Total**
	**Cat. 1**	0[a]	1	0	0	1	0	2
	**Cat. 2**	1	7[a]	4	4	2	1	19
	**Cat. 3**	0	9	33[a]	31	3	2	78
	**Cat. 4**	1	8	87	166[a]	32	15	309
	**Cat. 5**	0	5	33	73	48[a]	13	172
	**Cat. 5 or NC[b]**	1	15	165	314	135[a]	42[a]	672
	**NC**	0	9	80	182	104	92[a]	467
**Total**		3	54	402	770	325	165	1,719

a: Indicates where a correct prediction is made

b: Where chemicals were identified as > 2,000 mg/kg they were placed in category "Cat. 5 or NC" and not in Cat.5 or NC

c: Not including inconclusive predictions

Table S5: Breakdown of the results across different categories for the expert rule-based model

			Fit-for-purpose		Accuracy	
**Experimental value**	**Count**	**Number of inconclusive predictions[a]**	**Percentage correct or more conservative**	**Percentage correct or one more conservative**	**Percentage correct**	**Percentage correct (+/- one category)**
**Cat. 1**	2	0	0%	0%	0%	50.0%
**Cat. 2**	20	1	42.1%	42.1%	36.8%	63.2%
**Cat. 3**	80	2	53.9%	53.9%	42.3%	93.6%
**Cat. 4**	322	13	84.8%	81.9%	53.7%	92.2%
**Cat. 5**	182	10	92.4%	70.4%	27.9%	77.9%
**Cat. 5 or NC**	703	31	100.0%	73.1%	26.3%	73.2%
**NC**	496	29	100.0%	42.0%	19.7%	42.0%

a. Not included in the statistics

Table S6: Balanced summary statistics result for the expert rule-based model

	Fit-for-purpose		Accuracy	
	Average[a] percentage correct or more conservative	Average[a] percentage correct or one more conservative	Average[a] percentage correct	Average[a] percentage correct (+/- one category)
Expert rule-based model	74.6%	49.6%	28.7%	71.1%

a. Averages across all experimental classes, excluding compounds in the "Cat. 5 or NC" class and cat. 1 since there were only two chemicals which is not sufficient for a robust estimation.

c. Performance of the different methodologies

Table S7: Summary statistics showing the performance of the different methodologies

	Statistical-based method	Expert rule-based method	Consensus method
**Number of chemicals**	1,805	1,805	1,805
**Fit-for-purpose**			
Correct or more conservative	90.4%	93.7%	94.8%
Correct or one more conservative	73.4%	64.6%	61.6%
**Accuracy**			
Correct	45.7%	30.5%	27.0%
Correct (+/- one category)	80.8%	69.3%	66.1%
**Inconclusive**	18.2%	4.8%	5.4%

d. Consensus model for chemicals from the pharmaceutical sector

Table S8: Table showing counts of how the consensus model predicts for the different GHS categories from the pharmaceutical sector

		Predicted[c]						
**Experimental**		**Cat. 1**	**Cat. 2**	**Cat. 3**	**Cat. 4**	**Cat. 5**	**NC**	**Total**
	**Cat. 1**	0[a]	1	0	0	1	0	2
	**Cat. 2**	0	4[a]	2	2	2	1	11
	**Cat. 3**	1	3	22[a]	25	0	0	51
	**Cat. 4**	0	14	58	109[a]	15	2	198
	**Cat. 5**	0	2	21	23	10[a]	1	57
	**Cat. 5 or NC[b]**	1	27	105	202	63[a]	9[a]	407
	**NC**	0	7	39	67	16	19[a]	148
**Total**		2	58	247	428	107	32	874

a: Indicates where a correct prediction is made

b: Where chemicals were identified as > 2,000 mg/kg they were placed in category “Cat. 5 or NC” and not in Cat.5 or NC

c: Not including inconclusive predictions

Table S9: Breakdown of the results across different categories for the consensus model from the pharmaceutical sector

			Fit-for-purpose		Accuracy	
**Experimental value**	**Count**	**Number of inconclusive predictions[a]**	**Percentage correct or more conservative**	**Percentage correct or one more conservative**	**Percentage correct**	**Percentage correct (+/- one category)**
**Cat. 1**	2	0	0%	0%	0%	50.0%
**Cat. 2**	12	1	36.4%	36.4%	36.4%	54.6%
**Cat. 3**	53	2	51.0%	49.0%	43.1%	98.0%
**Cat. 4**	206	8	91.4%	84.3%	55.1%	91.9%
**Cat. 5**	61	4	98.3%	57.9%	17.5%	59.7%
**Cat. 5 or NC**	434	27	100.0%	67.3%	17.7%	73.2%
**NC**	158	10	100.0%	23.7%	12.8%	23.7%

a. Not included in the statistics

Table S10: Balanced summary statistics result for the consensus model from the pharmaceutical sector

	Fit-for-purpose		Accuracy	
	**Average[a] percentage correct or more conservative**	**Average[a] percentage correct or one more conservative**	**Average[a] percentage correct**	**Average[a] percentage correct (+/- one category)**
Consensus model	75.4%	50.3%	33.0%	65.6%

a. Averages across all experimental classes, excluding compounds in the "Cat. 5 or NC" class and cat. 1 since there were only two chemicals which is not sufficient for a robust estimation.

e. Consensus model for chemicals from the plant protection product sector

Table S11: Table showing counts of how the consensus model predicts for the different GHS categories from the plant protection product sector

		**Predicted[c]**						
**Experimental**		**Cat. 1**	**Cat. 2**	**Cat. 3**	**Cat. 4**	**Cat. 5**	**NC**	**Total**
	**Cat. 1**	0[a]	0	0	0	0	0	0
	**Cat. 2**	1	2[a]	2	0	0	0	5
	**Cat. 3**	0	6	5[a]	2	1	1	15
	**Cat. 4**	1	8	23	42[a]	4	0	78
	**Cat. 5**	1	3	6	21	3[a]	0	34
	**Cat. 5 or NC[b]**	2	19	48	112	37[a]	9[a]	227
	**NC**	3	5	13	41	11	3[a]	76
**Total**		8	43	97	218	56	13	435

a: Indicates where a correct prediction is made

b: Where chemicals were identified as > 2,000 mg/kg they were placed in category "Cat. 5 or NC" and not in Cat.5 or NC

c: Not including inconclusive predictions

Table S12: Table showing counts of how the consensus model predicts for the different GHS categories from the marketed plant protection product sector compounds that were retrieved from the Pesticide Properties Database

		Predicted[c]						
Experimental		Cat. 1	Cat. 2	Cat. 3	Cat. 4	Cat. 5	NC	Total
	Cat. 1	0[a]	0	0	0	0	0	0
	Cat. 2	0	0[a]	1	0	0	0	1
	Cat. 3	0	2	0[a]	0	1	0	3
	Cat. 4	0	1	2	2[a]	2	0	7
	Cat. 5	0	0	1	3	0[a]	0	4
	Cat. 5 or NC[b]	0	1	4	5	0[a]	0[a]	10
	NC	1	2	6	13	1	0[a]	23
Total		1	6	14	23	4	0	48

a: Indicates where a correct prediction is made

b: Where chemicals were identified as > 2,000 mg/kg they were placed in category "Cat. 5 or NC" and not in Cat.5 or NC

c: Not including inconclusive predictions

Table S13: Table showing counts of how the consensus model predicts for the different GHS categories from the other plant protection product sector proprietary / marketed compounds

		Predicted[c]						
**Experimental**		**Cat. 1**	**Cat. 2**	**Cat. 3**	**Cat. 4**	**Cat. 5**	**NC**	**Total**
	**Cat. 1**	0[a]	0	0	0	0	0	0
	**Cat. 2**	1	2[a]	1	0	0	0	4
	**Cat. 3**	0	4	5[a]	2	0	1	12
	**Cat. 4**	1	7	21	40[a]	2	0	71
	**Cat. 5**	1	3	5	18	3[a]	0	30
	**Cat. 5 or NC[b]**	2	18	44	107	37[a]	9[a]	217
	**NC**	2	3	7	28	10	3[a]	53
**Total**		7	37	83	195	52	13	387

a: Indicates where a correct prediction is made

b: Where chemicals were identified as > 2,000 mg/kg they were placed in category “Cat. 5 or NC” and not in Cat.5 or NC

c: Not including inconclusive predictions

Table S14: Breakdown of the results across different categories for the consensus model from the plant protection product sector

			Fit-for-purpose		Accuracy	
**Experimental value**	**Count**	**Number of inconclusive predictions[a]**	**Percentage correct or more conservative**	**Percentage correct or one more conservative**	**Percentage correct**	**Percentage correct (+/- one category)**
**Cat. 1**	0	0	N/A	N/A	N/A	N/A
**Cat. 2**	5	0	60.0%	60.0%	40.0%	100.0%
**Cat. 3**	15	0	73.3%	73.3%	33.3%	86.7%
**Cat. 4**	81	3	94.9%	83.3%	53.9%	88.5%
**Cat. 5**	37	3	100.0%	70.6%	8.8%	70.6%
**Cat. 5 or NC**	239	12	100.0%	69.6%	20.3%	69.6%
**NC**	85	9	100.0%	18.4%	4.0%	18.4%

a. Not included in the statistics

Table S15: Balanced summary statistics result for the consensus model from the plant protection products sector

	Fit-for-purpose		Accuracy	
	Average[a] percentage correct or more conservative	Average[a] percentage correct or one more conservative	Average[a] percentage correct	Average[a] percentage correct (+/- one category)
Consensus model	85.6%	61.1%	28.0%	72.8%

a. Averages across all experimental classes, excluding compounds in the "Cat. 5 or NC" class and cat. 1 since there were only two chemicals which is not sufficient for a robust estimation.

f. Consensus model for chemicals from the other chemical sector

Table S16: Table showing counts of how the consensus model predicts for the different GHS categories from the other chemical sectors

		Predicted[c]						
**Experimental**		**Cat. 1**	**Cat. 2**	**Cat. 3**	**Cat. 4**	**Cat. 5**	**NC**	**Total**
	**Cat. 1**	0[a]	0	0	0	0	0	0
	**Cat. 2**	0	2[a]	1	0	0	0	3
	**Cat. 3**	0	4	3[a]	4	1	0	12
	**Cat. 4**	1	2	7	12[a]	11	1	34
	**Cat. 5**	0	6	10	29	30[a]	8	83
	**Cat. 5 or NC[b]**	0	0	4	5	19[a]	1[a]	29
	**NC**	5	6	22	77	70	57[a]	237
**Total**		6	20	47	127	131	67	398

a: Indicates where a correct prediction is made

b: Where chemicals were identified as > 2,000 mg/kg they were placed in category “Cat. 5 or NC” and not in Cat.5 or NC

c: Not including inconclusive predictions

Table S17: Breakdown of the results across different categories for the consensus model from the other chemical sectors

			Fit-for-purpose		Accuracy	
**Experimental value**	**Count**	**Number of inconclusive predictions[a]**	**Percentage correct or more conservative**	**Percentage correct or one more conservative**	**Percentage correct**	**Percentage correct (+/- one category)**
**Cat. 1**	0	0	N/A	N/A	N/A	N/A
**Cat. 2**	3	0	66.7%	66.7%	66.7%	100%
**Cat. 3**	12	0	58.3%	58.3%	25.0%	91.7%
**Cat. 4**	35	1	64.7%	55.9%	35.3%	88.2%
**Cat. 5**	84	1	90.4%	71.1%	36.1%	80.7%
**Cat. 5 or NC**	30	1	100%	86.2%	69.0%	73.2%
**NC**	253	16	100%	53.6%	24.1%	53.6%

a. Not included in the statistics

Table S18: Balanced summary statistics result for the consensus model from other chemical sectors

	Fit-for-purpose		Accuracy	
	Average[a] percentage correct or more conservative	Average[a] percentage correct or one more conservative	Average[a] percentage correct	Average[a] percentage correct (+/- one category)
Consensus model	76.0%	61.1%	37.4%	82.8%

a. Averages across all experimental classes, excluding compounds in the "Cat. 5 or NC" class and cat. 1 since there were only two chemicals which is not sufficient for a robust estimation.

g. Consensus model across different sources of data

Table S19: Summary statistics showing the performance based on the consensus model across different sources of data

	All chemicals	Pharmaceutical sector	Plant protection product sector	Other chemical sector
**Number of chemicals**	1,805	926	462	417
**Fit-for-purpose**				
Correct or more conservative[a]	94.8%	94.1%	97.7%	93.4%
Correct or one more conservative[a]	61.6%	61.6%	63.2%	59.8%
**Accuracy**				
Correct[a]	27.0%	27.0%	23.2%	31.2%
Correct (+/- one category)[a]	66.1%	66.6%	65.1%	66.1%
**Inconclusive**	5.4%	5.6%	5.8%	4.6%

a. This excludes compounds with inconclusive predictions.

h. Expert review

Table S20: An assessment of incorrect consensus predictions (not including more conservative predictions) for experimental *in vivo* GHS category 1 and 2 chemicals from pharmaceutical companies

ID	Experimental GHS Classification	Predicted GHS Classification	Other information to support an expert review	Potentially corrected with expert review	Comments
**201**	Cat. 1	Cat. 2	API – anticoagulant	Yes	Knowledge of this mode of action (MoA) would support the Cat. 1
**23**	Cat. 1	Cat. 5	API - microtubule stabilizer	Yes	Knowledge of this MoA would support the Cat. 1
**185**	Cat. 2	Cat. 3	API – neuroscience	Yes	The MoA suggests a higher potency value
**209**	Cat. 2	Cat. 3	API – oncology	Yes	The MoA would support a category 1-3 assignment
**516**	Cat. 2	Cat. 4	This chemical is a starting material, it is Ames negative and borderline corrosive (OECD 431 neg at 3 min, borderline corrosive at 1h); the findings in the acute oral	Maybe	From a practical perspective, since this chemical would be classified as a dangerous good because of its corrosivity, an acute test may not be needed.

			study were related to corrosivity		
**2521**	Cat. 2	Cat. 4	This material is also classified as Acute Tox. 4 (harmful in contact with skin); Skin Sens. 1 (may cause an allergic skin reaction); Eye irritation 2 (causes serious eye irritation)	Yes	The MoA would support a category 1-3 assignment (CNS toxicity of related quinoline drugs reported).
**6**	Cat. 2	Cat. 5	API - DNA crosslinker	Yes	Knowledge of this MoA would support an assignment to Cat. 1
**207**	Cat. 2	Cat. 5	API - microtubule stabilizer	Yes	Knowledge of this MoA would support a more conservative GHS category
**5**	Cat. 2	NC	API – oncology drug (DNA/RNA alkylator)	Yes	The MoA would support a category 1-3 assignment

API – Active Pharmaceutical Ingredient; CNS – Central Nervous System; MoA – Mode of Action

Table S21: An assessment of incorrect consensus predictions (not including more conservative predictions) for experimental in vivo GHS category 1 and 2 chemicals from plant protection product companies

ID	Experimental GHS Classification	Predicted GHS Classification	Other information to support an expert review	Potentially corrected with expert review	Comments
**1427**	Cat. 2	Cat. 3	Close analogs in the database are assigned to cat. 3	No	
**2072**	Cat. 2[a]	Cat. 3	No additional information is available	No	

a The chemical is borderline cat. 2 and cat. 3

Table S22: An assessment of incorrect consensus predictions (not including more conservative predictions) for an experimental vivo GHS category 2 chemical from the other chemical companies

ID	Experimental GHS Classification	Predicted GHS Classification	Other information to support an expert review	Potentially corrected with expert review	Comments
703	Cat. 2	Cat. 3	The substance is a reactive fluorinated substance that would most commonly be used as an intermediate. In addition to Acute Oral Lethality Cat 2 it also has the following GHS Classifications:<br>• Acute Inhalation Toxicity Category 1<br>• STOT SE Cat 1 (Neurological and Respiratory)	Maybe	Without any close structural analogs with data in-house, the estimate would be reconsidered based on an expert review due to the inconsistent predictions (Cat 3 from one model and Cat 4 from another) and the lack of data from analogs.

DOI of original article: https://doi.org/10.1016/j.yrtph.2020.104843
Corresponding author. Present address: Instem, 1393 Dublin Road, Columbus, OH, 43,215, USA.
E-mail address: glenn.myatt@instem.com (G.J. Myatt).
